# Are drug consumption facilities an effective intervention to reduce drug related mortality? A critical review of the literature

**DOI:** 10.1192/bjo.2021.726

**Published:** 2021-06-18

**Authors:** Holly Melvin

**Affiliations:** University of Manchester

## Abstract

**Aims:**

To critically appraise the literature regarding the effect of Drug Consumption Facilities in reducing overdose mortality

Drug consumption facilities (DCF) are places where people can use illicit drugs in the presence of medically trained staff, they aim to reduce fatal overdose risk, reduce risky injecting practices, and to serve as a bridge for users into mainstream treatment, healthcare and social services. Increasing numbers of fatal overdoses due to illicit drug use are a significant public health concern. The UK's statutory independent advisory body (the Advisory Council on the Misuse of Drugs) has recommended DCFs as a mechanism to reduce fatal overdoses due to illicit drugs. However, current UK legislation prohibits their provision.

**Method:**

Systematic extraction of relevant literature from PubMed, using a search string with a focus on observational cohort studies with fatal overdose as the outcome. Appraisal of identified papers using the CASP tool

**Result:**

184 papers were identified, two of these met the inclusion criteria. Quality was fair/good. Neither demonstrated a clear effect of DCFs in reducing overdose mortality

**Conclusion:**

It is difficult to draw firm conclusions due to design weaknesses and potential confounding variables. Robust design is difficult in this research area, due to lack of suitability for RCTs. Despite the lack of a clear effect on overdose mortality, DCFs may exert other positive effects and are a pragmatic and humane response to reducing risk in this target population

